# Comprehensive analysis of germline mutations in northern Brazil: a panel of 16 genes for hereditary cancer-predisposing syndrome investigation

**DOI:** 10.1186/s12885-021-08089-9

**Published:** 2021-04-07

**Authors:** Amanda Ferreira Vidal, Rafaella Sousa Ferraz, Antonette El-Husny, Caio Santos Silva, Tatiana Vinasco-Sandoval, Leandro Magalhães, Milene Raiol-Moraes, Williams Fernandes Barra, Cynthia Lara Brito Lins Pereira, Paulo Pimentel de Assumpção, Leonardo Miranda de Brito, Ricardo Assunção Vialle, Sidney Santos, Ândrea Ribeiro-dos-Santos, André M. Ribeiro-dos-Santos

**Affiliations:** 1 Laboratory of Human and Medical Genetics, Graduate Program Genetics and Molecular Biology, Federal University of Pará, Belém, Pará Brazil; 2 Bettina Ferro de Souza University Hospital, Federal University of Pará, Belém, Pará Brazil; 3 João de Barros Barreto University Hospital, Federal University of Pará, Belém, Pará Brazil; 4 Center of Oncology Research, Federal University of Pará, Belém, Pará Brazil

**Keywords:** Hereditary cancer, Next generation sequencing, Pan-cancer panel, Pathogenic variant

## Abstract

**Background:**

Next generation sequencing (NGS) has been a handy tool in clinical practice, mainly due to its efficiency and cost-effectiveness. It has been widely used in genetic diagnosis of several inherited diseases, and, in clinical oncology, it may enhance the discovery of new susceptibility genes and enable individualized care of cancer patients. In this context, we explored a pan-cancer panel in the investigation of germline variants in Brazilian patients presenting clinical criteria for hereditary cancer syndromes or familial history.

**Methods:**

Seventy-one individuals diagnosed or with familial history of hereditary cancer syndromes were submitted to custom pan-cancer panel including 16 high and moderate penetrance genes previously associated with hereditary cancer syndromes (*APC, BRCA1, BRCA2, CDH1, CDKN2A, CHEK2, MSH2, MSH6, MUTYH, PTEN, RB1, RET, TP53, VHL, XPA* and *XPC*). All pathogenic variants were validated by Sanger sequencing.

**Results:**

We identified a total of eight pathogenic variants among 12 of 71 individuals (16.9%). Among the mutation-positive subjects, 50% were diagnosed with breast cancer and had mutations in *BRCA1*, *CDH1* and *MUTYH*. Notably, 33.3% were individuals diagnosed with polyposis or who had family cases and harbored pathogenic mutations in *APC* and *MUTYH*. The remaining individuals (16.7%) were gastric cancer patients with pathogenic variants in *CDH1* and *MSH2*. Overall, 54 (76.05%) individuals presented at least one variant uncertain significance (VUS), totalizing 81 VUS. Of these, seven were predicted to have disease-causing potential.

**Conclusion:**

Overall, analysis of all these genes in NGS-panel allowed the identification not only of pathogenic variants related to hereditary cancer syndromes but also of some VUS that need further clinical and molecular investigations. The results obtained in this study had a significant impact on patients and their relatives since it allowed genetic counselling and personalized management decisions.

**Supplementary Information:**

The online version contains supplementary material available at 10.1186/s12885-021-08089-9.

## Background

Hereditary cancer syndrome is a genetic predisposition to several types of cancer caused by pathogenic germline variants in one or more genes [1]. It corresponds to 5–10% of all cancers and have peculiar clinical aspects, such as the onset at an early age, high lifetime risk for multiple primary tumors, and cancer occurring in successive generations of the family [2, 3].

Most hereditary cancer syndromes display autosomal dominant inheritance involving genes that are mainly controlling cell cycle or DNA repair [4]. For example, mutations in *BRCA1* and *BRCA2* confer 40–80% lifetime risk of developing breast cancer, and 11–50% of developing ovarian cancer [5]; germline variants in at least one of the DNA mismatch repair genes (eg. *MSH2, MLH1, MSH6,* and *PMS1*) affect in up to 80% of the Lynch syndrome individuals [6]; and mutations in *CDH1* gene are detected in 30 to 40% of families with hereditary diffuse gastric cancer (HDGC) [7].

For a long time, single-gene analyses were used for detection of the genetic cause of hereditary cancers. However, the recent advances in DNA sequencing techniques have allowed the application of next generation sequencing (NGS) in clinical practice, including in genetic diagnosis of inheritance disease. In addition to its rapid, efficient and cost-effective approach for testing multiple cancer susceptibility genes, it may enhance the discovery of new susceptibility genes and enable individualized care of cancer patients [8, 9].

Most genomic cancer studies are carried out in North America and Western Europe, which do not fully represent the admixed Brazilian genetic background [8]. Currently, Brazilian heritability of cancer risk has been widely explored, so further studies might allow the development of more reliable surveillance approaches and medical guidelines for subjects carrying pathogenic germline mutations. In this context, we built an NGS custom pan-cancer panel containing 16 high and moderate penetrance genes previously associated with hereditary cancer syndromes (*APC, BRCA1, BRCA2, CDH1, CDKN2A, CHEK2, MSH2, MSH6, MUTYH, PTEN, RB1, RET, TP53, VHL, XPA* and *XPC*) [10, 11]. Here, we report the application of this panel in the investigation of such germline variants in patients from Northern Brazil presenting clinical criteria for hereditary cancer syndromes or familial history.

## Methods

### Participants

This study included 71 individuals diagnosed or with familial history of hereditary cancer syndromes (hereditary breast and ovarian cancer – HBOC, hereditary diffuse gastric cancer, Lynch syndrome, familial adenomatous polyposis or MUTYH-associated polyposis), according to National Comprehensive Cancer Network (NCCN) Clinical Practice Guidelines in Oncology. They were recruited from João de Barros Barreto University Hospital (HUJBB, Belém, Pará, Brazil). The study was approved by the Institutional Review Board from HUJBB (CAAE: 89363618.3.0000.5634), and all participants gave their written informed consent. The patients were selected according to the clinical criteria for hereditary syndrome according to National Comprehensive Cancer Network (NCCN) Clinical Practice Guidelines in Oncology. Individuals who did not meet the clinical criteria for hereditary syndromes were excluded.

### Next-generation sequencing

A custom pan-cancer panel was built containing 16 genes related to hereditary cancer syndromes (*APC, BRCA1, BRCA2, CDH1, CDKN2A, CHEK2, MSH2, MSH6, MUTYH, PTEN, RB1, RET, TP53, VHL, XPA* and *XPC*) [10]. Genomic DNA from the participants were extracted from whole blood samples collected during medical interview. DNA samples were quantified using Qubit 2.0 Fluorometer (Thermo Fisher Scientific), normalized to 25 ng/μL and custom pan-cancer library preparation. Briefly, indexed paired-end libraries were synthesized using TruSeq Custom Amplicon Library Prep Kit v1.5 (Illumina) and sequenced on MiSeq sequencing systems (Illumina). The raw sequencing reads of all libraries were deposited at the European Nucleotide Archive [Accession number: PRJEB43823].

Raw sequencing data were first filtered to remove low-quality reads and contaminants using Trimmomatic v.0.36 [12]. Resulting reads were aligned to the human genome (hg19) (http://hgdownload.soe.ucsc.edu/goldenPath/hg19/bigZips/) using BWA (v.0.7, [13]), and then sorted and indexed using sambamba [14] and samtools [15]. Small variants were identified using Genome Analysis Tool Kit (GATK) v3.8–0 [16] as follows. Alignments were recalibrated using RealignerTargetCreator and IndelRealigner tools against Mills & 1000G Gold Standard Indels (GATK resource bundle) (https://console.cloud.google.com/storage/browser/gcp-public-data%2D%2Dbroad-references/hg19/v0) [17]. Bases were recalibrated using BaseRecalibrator and dbSNP Build 138 (https://www.ncbi.nlm.nih.gov/snp/) [18] and variants were called using HaplotypeCaller. Joint genotyping was then performed using GenotypeGVCFs and the resulting SNPs and INDELs were filtered with VariantFiltration (SNPFilter with filterExpression: “QD < 2.0 || FS > 60.0 || MQ < 40.0 || MQRankSum < -12.5 || ReadPosRankSum < -8.0”; and INDELFilter with filterExpression: “QD < 2.0 || FS > 200.0 || ReadPosRankSum < -20.0”). Finally, all variants were annotated with information from the dbNSFP database v3.5a (https://sites.google.com/site/jpopgen/dbNSFP) [19] using the snpEff v4.3 tool [20].

ClinVar database (https://www.ncbi.nlm.nih.gov/clinvar/) was used to determine the clinical significance of all reported variants, in which variants were classified as pathogenic if they had a truncating effect. Pathogenicity of variants of uncertain significance (VUS) was investigated using ten different predictors: CADD [21], FATHMM [22], LRT [23], MetaSVM [24], MutationAssessor [25], MutationTaster [26], PROVEAN [27], Polyphen2 (HDIV and HVAR) [28] and SIFT [29], and those predicted by at least five predictors as likely pathogenic were considered to have a disease-causing potential. Variants were named according to Human Genome Variation Society nomenclature (HGVS, hpp://www.hgvs.org).

### Pathogenic variants validation

Pathogenic variants presence were validated by Sanger sequencing in ABI 3130 (Applied Biosystems) as follows. The location of interest was amplified by PCR using primers shown in Supplementary Table S1. Sanger Sequencing was carried out with 1 μL of purified PCR product of each exon, 0.5 μL of the forward/reverse specific primer, 0.5 μL of ABI Prism Bid Dye Terminator Cycle Sequencing v3.1 Kit (Applied Biosystems, USA), 3 μL of SaveMoney buffer, and 10 μL of water to a final volume of 15 μL. The thermocycling reaction proceeded as follows: 96 °C for 1 min, followed by 35 cycles of 96 °C for 15 s, 50 °C for 15 s and 60 °C for 4 min. The sequence information was interpreted by ABI Analysis Software™. The electropherograms were analyzed using the Chromas 2.6.6 software and compared with the reference sequence obtained from NCBI.

## Results

### Pan-cancer panel

We analyzed data from 71 individuals, including 60 cancer patients - breast cancer (68.3%), gastric cancer (16.7%), and other types of cancer (15%) - and 11 cancer-free individuals with family history of cancer, mainly familial adenomatous polyposis (FAP) (81.8%) (Table 1).

**Table 1:** Clinical characteristics of the individuals tested by the pan-cancer panel

Clinical Information	
**Number of patients**	
Affected patients	60 (84,5%)
Unaffected individuals with familial history of cancer	11 (15,5%)
**Sex**	
Female	61 (85,9%)
Male	10 (14,1%)
**Cancer types among affected patients**	
Breast cancer	41 (68,3%)
Gastric cancer	10 (16,7%)
Ovarian cancer	2 (3,3%)
Others^a^	7 (11,7%)
**Family history of unaffected individuals**	
FAP	9 (81,8%)
Breast cancer	1 (9,1%)
Adrenal cancer	1 (9,1%)

^a^Others: colorectal cancer (1), familial adenomatous polyposis (FAP) (1), colonic polyps (1), glioblastoma (1), kidney cancer (1), adrenal cancer (1) and both kidney and prostate cancer (1)

A total of eight pathogenic (either pathogenic or likely pathogenic) variants were identified in *APC, BRCA1, CDH1, MSH2* and *MUTYH* among 12 mutation-positive individuals (16.9%). Insertions and deletions represented 50% (*n* = 4) of all pathogenic variants, whereas single nucleotides variants (SNVs) accounted for the other half. The functional consequences of the identified pathogenic variants were primarily frameshift effects (50%), followed by stop gained (37.5%) and missense (12.5%) (Table 2), all of them were confirmed by Sanger sequencing. No variants described as pathogenic were identified in 11 genes (*BRCA2, CDKN2A, CHEK2, MSH6, PTEN, RB1, RET, TP53, VHL, XPA* and *XPC*).

**Table 2:** Pathogenic variants identified in individuals affected with cancer and unaffected individuals with cancer familial history

Gene	Exon	rsID	HGVS_DNA	HGVS_ protein	Type of mutation	Impact	Cancer history (n° probands)
*APC*	16	–	c.2195dupA	p.Asn732fs	Frameshift	High	FAP (1) and family history of FAP (2)
*BRCA1*	10	rs80357296	c.3544C > T	p.Gln1182*	Stop gained	High	Breast cancer (1)
*BRCA1*	10	rs80357522	c.1961delA	p.Lys654fs	Frameshift	High	Breast cancer (2)
*CDH1*	7	rs587780784	c.1003C > T	p.Arg335*	Stop gained	High	Breast cancer (1)
*CDH1*	8	rs587776398	c.1023 T > G	p.Tyr341*	Stop gained	High	Gastric cancer (1)
*MSH2*	3	rs63750704	c.388_389delCA	p.Gln130fs	Frameshift	High	Gastric cancer(1)
*MUTYH*	13	rs36053993	c.1187G > A	p.Gly396Asp	Missense	Moderate	Breast cancer (2) and colonic polyps (1)
*MUTYH*	12	rs587778536	c.1147delC	p.Ala385Profs	Frameshift	High	Colonic polyps (1)

*FAP* familial adenomatous polyposis, Human Genome Variation Society (HGVS), rsID in dbSNP (rsID)

Most of these pathogenic mutations identified were nonrecurrent (62.5%). Among the recurrent mutations, *MUTYH* c.1187 G > A (p.Gly396Asp) was reported in unrelated individuals, whereas *BRCA1* c.1961delA (p.Lys654fs) and *APC* c.2195dupA (p.Asn732fs) were reported in related individuals.

Almost all probands with positive results for pathogenic variants had only a single mutation (*n* = 11), with the exception of one proband affected with colonic polyps who was compound heterozygous for *MUTYH* (c.1187G > A and c.1147delC) in accordance with the recessive pattern of MUTYH-associated polyposis.

Among the individuals with pathogenic variants, 50% were diagnosed with breast cancer and had mutations in *BRCA1*, *CDH1* and *MUTYH* – gene not typically associated with this cancer. Notably, 33.3% were individuals diagnosed with polyposis or who had family cases and harbored pathogenic mutations in *APC* and *MUTYH*. The remaining individuals (16.7%) were gastric cancer patients with pathogenic variants in *CDH1* and *MSH2*.

**Fig. 1:**
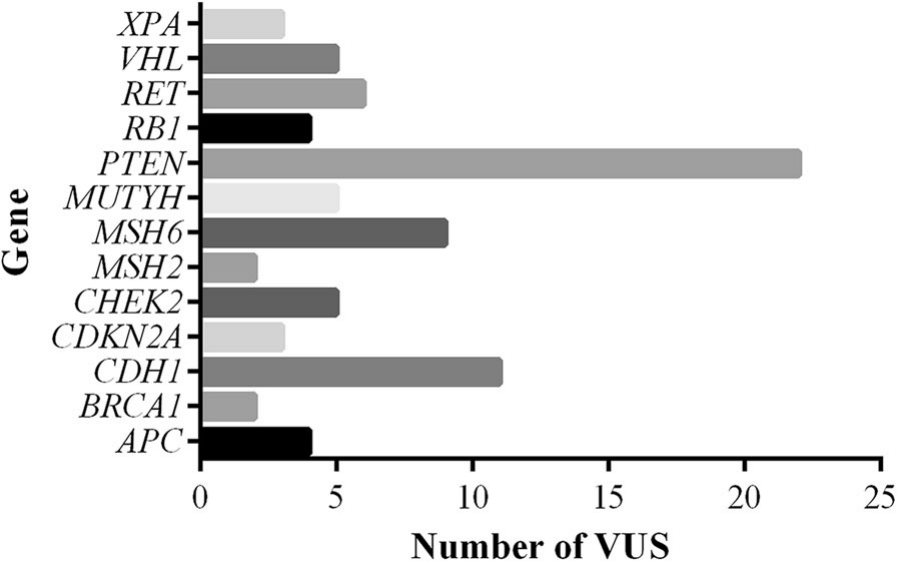
Count of variants of uncertain significance (VUS) by gene

A total of 81 VUS were identified in *APC, BRCA1, CDH1, CDKN2A, CHEK2, MSH2, MSH6, MUTYH, PTEN, RB1, RET, VHL* and *XPA. PTEN* presented the highest amount of VUS with 22 variants (Fig. 1). Among all individuals tested, 54 (76.05%) presented at least one VUS and 14 individuals had a negative result for both pathogenic variants and VUS.

Most VUS were in intronic, intragenic or untranslated regions (UTR) and, therefore, it was not possible to predict their pathogenicity (Supplementary Table S2). Only 12 variants were submitted to the prediction (Supplementary Table S3), among these seven were predicted to be deleterious by at least five predictors (six missense variants and one structural interaction variant), suggesting their disease-causing potential.

### Family screening

**Fig. 2:**
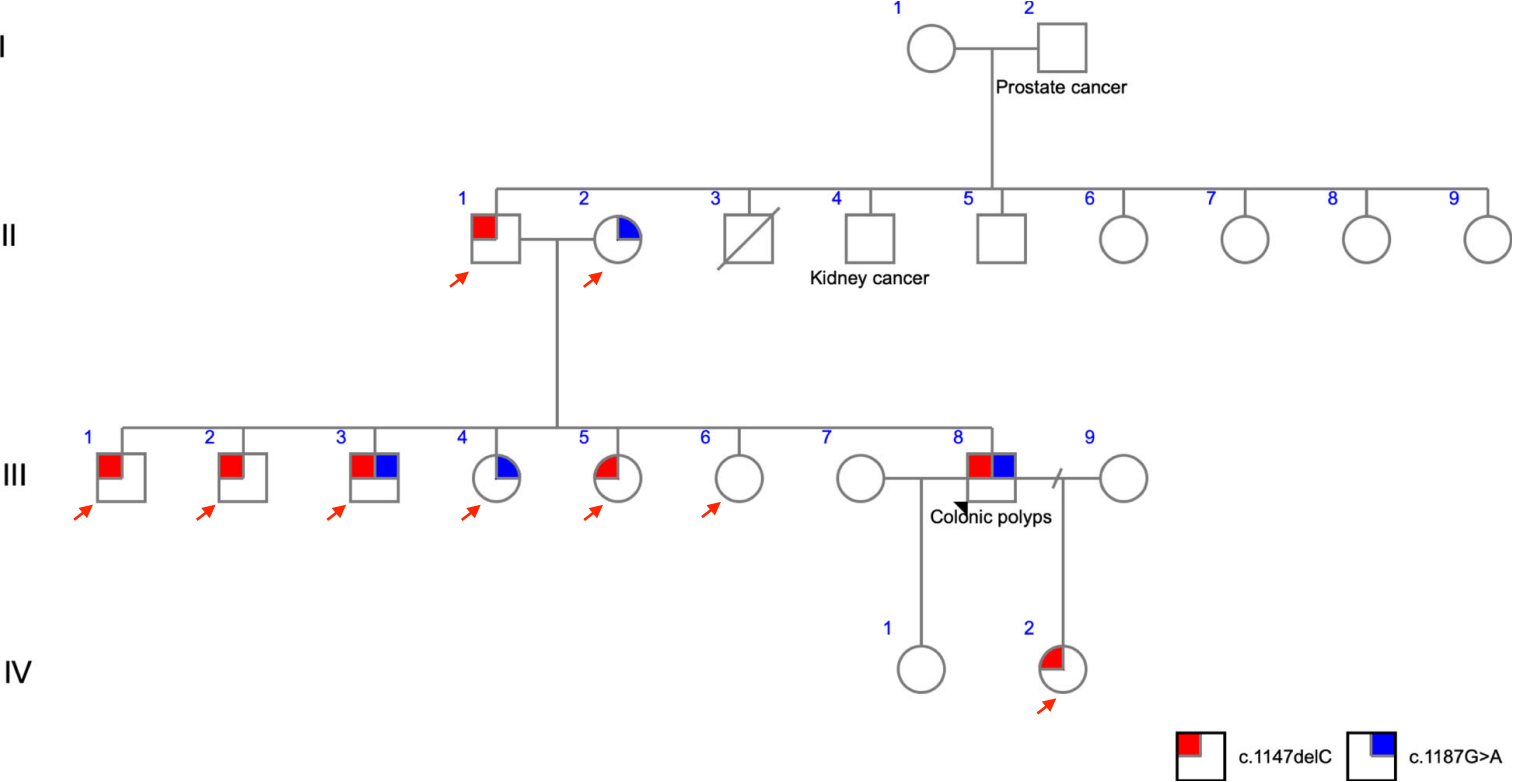
Pedigree of mutation-positive proband of family A. Black arrow indicates the proband with colonic polyps affected by two variants in *MUTYH* gene (c.1147delC and c.1187G > A) (III8). Red arrow indicates family members tested for mutations. A small red square indicates individuals carrying the mutation c.1147delC. A small blue square indicates individuals carrying the mutation c.1187G > A

**Fig. 3:**
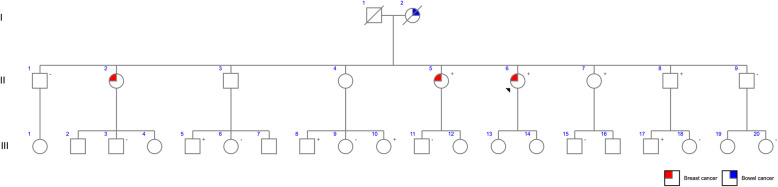
Pedigree of mutation-positive proband of family B. Black arrow indicates the proband carrying *BRCA1* c.1961delA mutation (II6). Small red square indicates individuals diagnosed with breast cancer. Small blue square indicates individuals diagnosed with bowel cancer. (+) represents individuals tested for mutation with *BRCA1* c.1961delA mutation. (−) represents individuals tested for mutation without *BRCA1* c.1961delA mutation

The pan-cancer panel allowed us to identify familial mutation in affected patients, directing screening on family members and, consequently, to provide a personalized genetic counselling and management decisions. Examples of families that were benefited from the pan-cancer panel results are depicted in Fig. 2 and Fig. 3.

Family A (Fig. 2) proband was submitted to genetic testing due to colonic polyposis diagnosis. Two pathogenic variants in *MUTYH* gene were identified, c.1147delC and c.1187G > A. Nine unaffected family members were investigated – of these, three men and two women presented c.1147delC, two women presented c.1187G > A, while one man was compound heterozygous for these variants. Family B (Fig. 3) proband was submitted to genetic testing since they met the clinical criteria for HBOC. This individual presented *BRCA1* c.1961delA. Sixteen family members were tested to this variant, being seven mutation-positive (four men and three women).

Therefore, as pathogenic variants were identified in unaffected individuals with an affected family member, it allowed the choosing of an appropriate surveillance approach and management decisions for the mutation-negative subjects.

## Discussion

Advances in NGS technologies and their cost-effectiveness have made multigene panels a useful diagnostic tool in oncology clinic, particularly for offering an increase in mutation detection rate, which benefits individuals without family history information or atypical phenotype [30]. In the present study, a custom pan-cancer panel was developed and applied for the detection of hereditary cancer-related pathogenic mutations.

Variant analysis revealed the presence of at least one pathogenic variant in 16.9% of the analyzed individuals. Most of the individuals were breast cancer patients, being *BRCA1* mutations responsible for 50% of the pathogenic variants in these patients. In the example of a family which was benefited from the multigene panel analysis, *BRCA1* c.1961delA was also reported in men, demonstrating the importance of *BRCA1* screening not only in women, since men with pathogenic variants in this gene have a slightly higher risk for prostate cancer and male breast cancer [31, 32].

Germline mutations in *BRCA1* and *BRCA2* are very relevant to the development of HBOC, however, they are responsible for only 15–25% of such cases, which demonstrates the significant contribution of other genes [33, 34]. In this study, the remaining pathogenic variants in breast cancer occurred in *CDH1* and *MUTYH*.

*CDH1* encodes E-cadherin, a protein responsible for calcium-dependent cell-to-cell adhesion. Germline mutations in this gene have been reported to cause hereditary diffuse gastric cancer (HDGC), a disorder that leads to an increased risk for diffuse gastric cancer and lobular breast cancer (LBC) [35]. However, substantial evidences have demonstrated an increased risk for LBC among *CDH1* mutations carriers regarding their familial history for diffuse gastric cancer (DGC) [36]. In this study, *CDH1* c.1003 C > T was reported in a breast cancer patient without familial history for DGC.

*MUTYH* mutations are the cause of MUTYH-associated polyposis (MAP), a syndrome that predisposes to colorectal polyposis and colorectal cancer [37]. Here, *MUTYH* c.1187G > A was reported in two unrelated individuals with breast cancer. This variant is the most frequent of all *MUTYH* mutations in various populations [38], but the association between this variant and breast cancer remains controversial. In Northern Israel, monoallelic inheritance was associated with an elevated risk of breast cancer [39], while in Non-Hispanic individuals of European ancestry there was no positive association [40]. Thus, our preliminary finding in the Brazilian population reinforces the need for further studies and for genetic counseling for families that have this variant segregating.

Here, one individual with colonic polyposis showed compound heterozygous mutations in *MUTYH* (c.1147delC and c.1187G > A)*.* Genetic counselling was essential to identify other biallelic carriers in the family, in addition to monoallelic carriers (potential increased breast cancer risk), since these individuals may have a 2-*fold* increased risk for colorectal cancer and other cancers when compared to the general population [41].

Among the individuals with gastric cancer, one presented a mutation in *CDH1* and, another, in *MSH2*. *CDH1* alterations underlie HDGC, conferring risk of 56–70% higher [42]. Gastric cancer occurrence has a frequency of 1–6% in individuals with a Lynch syndrome-associated mutation, and this risk increases by 9% for those that present germline *MSH2* mutations [43, 44]. In this study, a mutation in this gene was identified in a gastric cancer patient (c.388_389delCA) – until now, this variant had been reported only in Brazilian Lynch syndrome patients [45, 46].

Multigene pan-cancer panels that include a variety of cancer types might contribute to better understanding an individual’s risk for cancer, but it also raises new challenges in genetic counselling [9]. These challenges are even more complex in populations with a broad lack of information, such as the Brazilian one. Our population has several particular genomic features resulting from a high degree of admixture [47]. Most of the available genomic databases contain data from ancestry populations that do not entirely represent our genetic composition. It may explain the low number of pathogenic variants found in this study − it is possible that among the VUS and the “not described” variants there are some pathogenic variants that have not been associated with clinical data yet due to the lack of studies and validation [48]. Among the 81 VUS identified, seven were predicted with deleterious potential by at least five different predictors, reinforcing the need for functional studies that validate their pathogenicity.

Moreover, it is important to comprehend that most studies on cancer genetics were carried out in North America and Western Europe, thus reflecting the mutation burden in subjects of European ancestry. It is known that people of distinct ethnicities inherit a different pattern of pathogenic mutations from their ancestors. For instance, *BRCA1* and *BRCA2* show significant global variations according to contribution in regional cancer incidence and to mutation spectrum [49]. Only a minor part of the heritability of cancer risk has been elucidated so far, and further whole exome sequencing studies are needed to significantly increase the identification of hereditary cancer genes [8].

Despite the small sample size, which may not fully represent the Brazilian population, our results suggest the existence of a unique genetic background which needs to be more explored. Considering Brazil as a continental country submitted to different colonization processes, further studies should include samples from the different regions of the country.

## Conclusions

In conclusion, our findings contributes to the description of pathogenic variants background in Northern Brazil, as well as demonstrated the potential of a multigene panel in identifying pathogenic variants in genes not typically tested in hereditary cancer specific cases. The results obtained in this study observed 81 VUS, seven predicted with deleterious potential, reinforcing the need to identify pathogenicity of these variants in our population. These results had a great impact on the patients and their relatives since it allowed genetic counselling and personalized management decisions.

## Supplementary Information

**Additional file 1: Supplementary Table S1** Primers used for validation of pathogenic variants reported in the pan-cancer panel.

**Additional file 2: Supplementary Table S2** List of variants of uncertain significance (VUS) reported in pan-cancer panel.

**Additional file 3: Supplementary Table S3** Pathogenicity prediction of the variants of uncertain significance.

## Data Availability

The raw sequencing reads of all libraries were deposited at the European Nucleotide Archive [Accession number: PRJEB43823]. The datasets obtained from web-based sources and subsequently analysed in our study were: human genome (hg19) (http://hgdownload.soe.ucsc.edu/goldenPath/hg19/bigZips/), Mills & 1000G Gold Standard Indels (GATK resource bundle) (https://console.cloud.google.com/storage/browser/gcp-public-data%2D%2Dbroad-references/hg19/v0), dbSNP Build 138 (https://www.ncbi.nlm.nih.gov/snp/), dbNSFP database v3.5a (https://sites.google.com/site/jpopgen/dbNSFP) and ClinVar database (https://www.ncbi.nlm.nih.gov/clinvar/).

## Abbreviations

DGC: Diffuse gastric cancer
HBOC: Hereditary breast and ovarian cancer
HDGC: Hereditary diffuse gastric cancer
LBC: Lobular breast cancer
NGS: Next generation sequencing
SNVs: Single nucleotides variants
UTR: Untranslated regions
VUS: Variant uncertain significance
