# Supplementary material for: Comprehensive analysis of germline mutations in northern Brazil: a panel of 16 genes for hereditary cancer-predisposing syndrome investigation
Source: BMC Cancer. 2021 Apr 7;21:363. doi: 10.1186/s12885-021-08089-9 (PMC8028728; doi:10.1186/s12885-021-08089-9)
Supplement: Supplementary file 1 — Additional file 1: Supplementary Table S1 Primers used for validation of pathogenic variants reported in the pan-cancer panel. [file 12885_2021_8089_MOESM1_ESM.docx]

**Supplementary Table S1** Primers used for validation of pathogenic variants reported in the pan-cancer panel.

| **Gene** | **Variant** | **Primers(5’-3’)** |
| --- | --- | --- |
| *APC* | c.2195dupA | F: TGG AAC TTT GTG GAA TCT CTC A  R: TGC TTG TGT CTC TGC TTA CTA CG |
| *BRCA1* | c.3544C>T | F: GTA CTA ATG AAG TGG GCT CC  R: GTT CTT AGA CAG ACA CTC G |
| *BRCA1* | c.1961delA | F: CCT AAC CCA ATA GAA TCA CTC G  R: GGT ACC AAT GAA ATA CTG CTA C |
| *CDH1* | c.1003C>T | F: TTC TTT CTC CCC TAG CAC TTT G  R: ACA ACT GGC CTA GCA GGA TTT |
| *CDH1* | c.1023T>G | F: CTT GGT TGT GTC GAT CTC TCT G  R: GAC CTT TCT TTG GAA ACC CTC T |
| *MSH2* | c.388_389delCA | F: ATT GAA CCC TTG AGG CAG AG  R: TGC TTA CCT GTC TCA GTT TCC C |
| *MUTYH* | c.1187G>A | F: CTT GGC TTG AGT AGG GTT CG  R: GCT TGA TGT GAG AGA AGG TGT G |
| *MUTYH* | c.1147delC | F: CTT GGC TTG AGT AGG GTT CG  R: GCT TGA TGT GAG AGA AGG TGT G |

F: forward; R: reverse
